# Untargeted metabolomics tools to inform diet and drug exposures of cognitively declined cohorts

**DOI:** 10.1002/alz.089880

**Published:** 2025-01-09

**Authors:** Haoqi Nina Zhao, Ipsita Mohanty, Rima F. Kaddurah‐Daouk, Alzheimer's Gut Microbiome Project Consortium (AGMP), Pieter Dorrestein

**Affiliations:** ^1^ University of California ‐ San Diego, La Jolla, CA USA; ^2^ Skaggs School of Pharmacy and Pharmaaceutical Science, University of California‐San Diego, San Diego, CA USA; ^3^ Duke University, Durham, NC USA; ^4^ University of California, San Diego, La Jolla, CA USA

## Abstract

**Background:**

It is now widely acknowledged that diet, lifestyle, and environmental exposures largely affect an individual’s metabolic state in health and disease, including the brain. Metabolomics has demonstrated its potential to enable exciting discoveries in brain health, facilitated by advances in analytical and informatics techniques. Here, we highlighted the use of MS/MS‐based untargeted metabolomics to study the diet and medication exposure of cognitively declined cohorts through the newly developed FoodMASST and DrugMASST tools.

**Methods:**

A food metabolomics reference dataset was created with tandem mass spectrometry data (MS/MS) alongwith detailed and structured metadata for ∼3,500 foods. We used this resource to trace the food origin of important Alzheimer’s disease metabolite biomarkers as well as to investigate the empirical diet patterns of subjects in a Mediterranean diet intervention study (MIND). An MS/MS spectral library for drugs, metabolites, and related molecules was developed by spectral similarity search against ∼1.2 billion public metabolomics spectra. We utilized this resource to perform empirical medication read‐out in 501 brain samples from the Rush Religious Orders and Memory and Aging Project (ROS/MAP) and 637 fecal samples from the Aging and Disability Resource Centers (ADRCs).

**Results:**

We demonstrated the utility of the FoodMASST and DrugMASST tools through several examples. Using FoodMASST we demonstrated that lentil is an important food source of tryptophan betaine, an important AD marker. FoodMASST‐based diet score separated the metabolomics profiles of MIND subjects in the PCoA cohort. DrugMASST detected 102 drugs in brain samples from the ROS/MAP and revealed that neurology/psychiatry and cardiology drugs were the most important categories. The computationally derived drug metabolite library enabled additional annotation of 15 drug metabolites with high confidence. We observed significant separation in the metabolome of patients with and without antibiotic detection. In the ADRC fecal samples, we detected 190 drugs and the neurology/psychiatry and cardiology categories had the highest number of drugs observed. Carbidopa, a dopamine promoter drug given to treat PD, was detected in 62% of the patients.

**Conclusions:**

Untargeted metabolomics tools, such as metadata‐driven metabolomics exemplified by FoodMASST and DrugMASST, create insights into patients' chemical exposure and their role in gut microbiome and Alzheimer’s disease.

